# Comparison of Difficult Airway with Dental Malocclusions during Laryngoscopy with Video Laryngoscopy in Patients Undergoing Elective Surgery -

**DOI:** 10.31661/gmj.v14i.3586

**Published:** 2025-12-29

**Authors:** Mehrdad Malekshoar, Tayyebeh Zarei, Maryam Pourbahri, Mohammad Shirgir, Majid Vatankhah

**Affiliations:** ^1^ Anesthesiology, Critical Care and Pain Management Research Center, Hormozgan University of Medical Sciences, Bandar Abbas, Iran

**Keywords:** Airway, Dental Malocclusions, Laryngoscopy, Elective Surgery, Hormozgan

## Abstract

**Background:**

Managing difficult airways in surgical patients remains a significant challenge for primary care physicians and anesthesiologists, often leading to high-stress situations. Dental malocclusion is a critical factor that can complicate airway management during laryngoscopy.To investigate the relationship between dental malocclusion classes and difficult airway indicators, such as video laryngoscopy grade and Malampathi criteria, in patients undergoing elective surgery.

**Materials and Methods:**

This descriptive-analytical study randomly sampled patients scheduled for elective surgery at Shahid Mohammadi Hospital in 2022. Data were collected using a checklist that included variables such as age, sex, height, weight, dental malocclusion class, Malampathi scale, and video laryngoscopy grade. The Chi-square test and multiple logistic regression were applied to compare proportions between groups. A p-value of 0.05 was considered statistically significant.

**Results:**

The study found no significant relationship between dental malocclusion class and height, weight, or age. However, the prevalence of malocclusion across all three classes was higher in male patients. A significant association was observed between dental malocclusion class and both Malampathi criteria and video laryngoscopy grade (P0.005). Specifically, as the malocclusion class increased, both the laryngoscopy grade and Malampathi grade also increased.

**Conclusion:**

This study highlights a significant relationship between dental malocclusion class and difficult airway indicators, such as video laryngoscopy grade and Malampathi criteria. Higher grades of laryngoscopy grade and Malampathi criteria were associated with Class 2 and Class 3 dental malocclusions. These findings underscore the importance of dental occlusion assessment in predicting and managing difficult airways in surgical patients.

## Introduction

Malocclusion is defined as a disabling dentofacial anomaly. Specifically, the World Health Organization refers to it as an abnormal occlusion or disruption of craniofacial relationships that may affect appearance, beauty, function, facial coordination, and psychosocial health [1]. Moreover, improper oral habits can not only disturb the position of the teeth but also particularly affect the normal skeletal growth pattern [2]. To better understand malocclusion, in 1899, Edward Hartley Engel, known as the father of modern orthodontics, classified malocclusions into three classes (Class I, II, and III) based on the alignment of the permanent first molars and the relationship between the upper and lower jaws [3].

Among these classes, patients with retrognathic mandible in Class 2 malocclusion have the smallest oropharyngeal dimensions, whereas those with Class 3 malocclusion have the largest dimensions. Interestingly, no significant difference in airway orientation was found in patients with skeletal malocclusion. However, patients with Class 3 skeletal malocclusion tend to have a vertical airway orientation, in contrast to patients with Class 2 skeletal malocclusion, who exhibit a forward airway orientation. These differences in airway dimensions, combined with other structural issues, can lead to respiratory problems in these patients. Therefore, proper detection and early intervention are crucial for such patients [4].

In addition to respiratory concerns, difficult airway management poses a significant challenge in postoperative anesthesia care. To address this, several airway management techniques are available, including oral intubation, nasal intubation, blind nasal intubation, fiberoptic-guided nasal intubation, and tracheostomy [5]. In this context, anesthesiologists play a crucial role in securing the airway and providing initial resuscitation and stabilization for these patients. Thus, careful planning for airway management during elective surgery and in the postoperative period is essential [6].

One advanced tool for airway management is video laryngoscopy, which utilizes video camera technology to visualize airway anatomy and facilitate endotracheal intubation [7]. However, difficult direct laryngoscopy, which complicates tracheal intubation during anesthesia, remains a significant clinical concern. If not managed promptly and effectively, difficult laryngoscopy can increase the risk of airway injury, aspiration, hypoxic brain damage, and even death. In cases where anesthesia is not anticipated before induction and immediate management techniques fail, surgical procedures may need to be canceled or rescheduled to allow for further management [8].

Seo et al. revealed that the Upper Lip Bite Test (ULBT) is a strong indicator of difficult intubation. Specifically, a Class III ULBT, where the lower teeth are unable to bite the upper lip, showed an odds ratio of 12.48 for predicting difficult intubation. However, unlike the ULBT, buck teeth did not show a significant association with difficult intubation [9]. Another study showed that ULBT which evaluates the relationship between the upper lip and teeth, demonstrated greater sensitivity and specificity in predicting difficult laryngoscopy compared to other tests, such as the mouth opening test. In contrast, the modified Mallampati test, which assesses the visibility of the uvula and soft palate, showed a sensitivity of only 0.51 for predicting difficult tracheal intubation [10]. Based on a systematic review, while multiple tests can assist in detecting potentially difficult intubation, the most reliable predictor was the inability to bite the upper lip using the lower teeth [11]. The structure of the mandible (jawbone) and the alignment of teeth may influence the upper airway space, potentially contributing to breathing challenges and a higher likelihood of obstructive sleep apnea. Furthermore, ensuring upper airway openness during anesthesia is important. This can be facilitated by adjusting the position of the mandible, such as through jaw closure [12].

Existing research shows the challenges and limitations of managing difficult airways, especially in patients with dental malocclusions. However, there is a notable lack of studies comparing difficult airways in patients with dental occlusions during laryngoscopy versus video laryngoscopy. To address this gap, our study focuses on exploring this critical area to uncover potential predictors of airway complications. Additionally, we aim to investigate the connection between different classes of dental malocclusion and video laryngoscopy grades, as well as the Malampathi scale, which sets our research apart in the context of elective surgery. Therefore, we conducted a study to investigate the relationship between difficult airways and dental malocclusions.

## Materials and Methods

This study was a descriptive and analytical study conducted from 2021 to 2022 at Shahid Mohammadi Hospital, involving patients undergoing elective surgery. A simple random sampling method was used, where patients were selected randomly from the hospital's database using a table of random numbers to achieve the required sample size for each group.

Inclusion criteria: All patients undergoing elective surgery at Shahid Mohammadi Hospital in Bandar Abbas from 2021 to 2022.

Exclusion criteria: patients undergoing emergency surgery and those who did not provide informed consent to participate in the study.

The sample size was calculated using the ratio comparison formula for two independent groups, based on a similar study [13], which reported a prevalence of Class I malocclusion (P1=0.49) and Class II malocclusion (P2=0.42). Using G*Power software, with an alpha level of 0.05 (95% confidence interval) and 80% power, the calculated sample size for each group was 103.

The sample size calculation formula used was: n=((z_(1-α/2)+z_(1-β))^2 * (p1q1 + p2q2)) / (p1-p2)^2, which yielded a sample size of 103 per group.

Data were collected using a checklist, which included information from the patient's medical record and physical examination. The study variables included age, sex, height, weight, degree of dental malocclusion, and Mallampati classification.

Patients were examined by a trained dental student, under the supervision of an orthodontic specialist, to determine the type of dental malocclusion (Class I, II, or III) using natural light. The Mallampati classification (Class I, II, III) was used to assess the occlusion status of the patients.

After monitoring, patients received premedication with midazolam (0.03 mg/kg), fentanyl (2 micrograms/kg), and then induction with propofol (1-2 mg/kg) and atracurium (0.6 mg/kg). Patients were then intubated by an anesthesiologist, and the video laryngoscopy grade was recorded. The patient's head was placed in the sniffing position, and laryngoscopy was performed using a Macintosh Blade No. 3 laryngoscope. Then, intratracheal intubation was performed. The patient's laryngoscopy grade (Grade III) and Cormack-Lehane classification were evaluated. Patients with a Grade III Cormack-Lehane classification and a problematic laryngoscopy view were classified as having a difficult laryngoscopy.

If any of the following occurred, the patient was considered to have difficult intubation: use of a maneuver or special equipment (e.g., external laryngeal pressure, head repositioning, Macintosh blade No. 4), three or more attempts at laryngoscopy and intubation, or a Cormack-Lehane classification of Grade III or IV.

The collected data were summarized using statistical indicators and tables. Data analysis was performed using SPSS version 24 software (version 16, IBM Corp., Armonk, NY, USA), employing descriptive statistics (mean, standard deviation, frequency, and percentage) to summarize the data. Chi-square tests and multiple logistic regression analysis were used to compare the proportions between the two groups. A p-value of <0.05 was considered statistically significant in all analyses.

## Results

**Figure-1:**
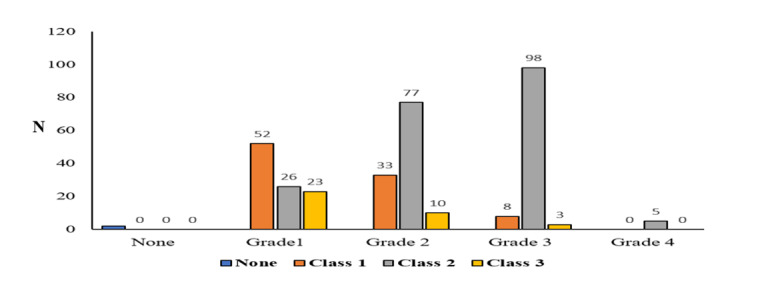
Figure 1. The results of the relationship between dental malocclusion and video laryngoscopy grade in patients with difficult airway.

**Figure-2:**
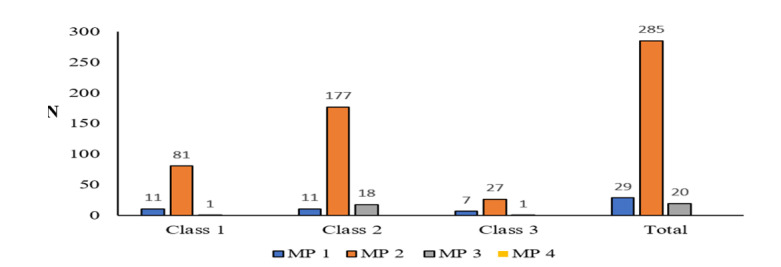
Figure 2. The results of the relationship between dental malocclusion and malampathi criteria in patients with difficult airway

**Table:** Table1. Characteristics of Subjects

Variable	Total	Class 1	Class 2	Class 3	P
Number	335	93	206	36	
Mean Age (years)	41.06±15.62	40.44±13.7	41.99±16.52	39.66±12.08	0.79
Mean Weight (kg)	69.97±13.33	69.47±15.45	69.24±10.53	69.94±12.45	0.51
Mean Height (cm)	170.25±16.85	170.22±14.3	171.12±8.62	169.25±10.01	0.57
Gender	Male	213 (63.2%)	64 (19%)	133 (35.5%)	16 (7.4%)	0.005
Female	122 (36.4%)	28 (8.3%)	73 (21.7%)	20 (5.9%)	

The study included a total of 335 subjects, divided into three classes: Class 1 (n=93), Class 2 (n=206), and Class 3 (n=36). The mean age of the participants was 41.06 ± 15.62 years, with no significant differences observed across the classes (P=0.79). Similarly, mean weight (69.97 ± 13.33 kg, P=0.51) and mean height (170.25 ± 16.85 cm, P=0.57) did not differ significantly among the groups. Gender distribution, however, showed significant variation (P=0.005). Males constituted 63.2% (n=213) of the total sample, with the majority in Class 2 (35.5%, n=133). Females accounted for 36.4% (n=122) of the sample, with the highest proportion in Class 2 (21.7%, n=73). However, a significant difference is observed in the distribution of males and females between classes (P=0.005), with Class 2 having the highest percentage of males (35.5%) and Class 3 having a higher percentage of females (5.9%) compared to Class 1, as shown in Table-1. Figure-1 shows the distribution of laryngoscopy grades among the 335 patients: Grade 1 (101 patients, 30.1%), Grade 2 (120 patients, 35.8%), Grade 3 (109 patients, 32.5%), and Grade 4 (5 patients, 1.5%). The results indicate a statistically significant association between the grade of video laryngoscopy and the class of dental malocclusion (P<0.005). Figure-1 shows that among patients with Grade 3 and 4 laryngoscopy, Class 2 dental malocclusion is the most common. Furthermore, among patients with Grade 1 laryngoscopy, Class 1 dental malocclusion was the most frequent, with 52 patients (15.4%). Figure-2 shows the distribution of Malampati scores among the 335 patients with dental malocclusion: 285 patients (85.1%) had a Malampati score of 2, 29 patients (8.7%) had a score of 1, and 20 patients (6%) had a score of 3. The evaluation based on the Malampati criterion revealed a statistically significant difference in dental occlusion class according to the Malampati score (P<0.005).

## Discussion

This study investigated the relationship between dental malocclusion classification and laryngoscopy grade to predict difficult intubation. Airway management is critical for anesthesiologists, typically performed via tracheal intubation under direct laryngoscopy. However, difficult intubation can lead to various complications, ranging from minor issues like sore throat to more serious injuries [14]. To address this challenge, most studies on airway examination rely on anatomical signs and non-invasive clinical methods. For instance, clinical criteria for airway assessment before anesthesia induction include Mallampati classification, mouth opening size, thyromental distance, mandibular length, neck extension, sternomental distance, and other methods such as evaluating dentofacial abnormalities [15]. In this context, laryngoscopy was categorized as easy (Grades 1 and 2) or difficult (Grades 3 and 4). This study focuses on dentofacial anomalies, particularly malocclusion, as a potential predictor of difficult laryngoscopy. Malocclusion is associated with a significant challenge: difficult intubation. Therefore, anticipating difficult intubation through such predictors can help mitigate anesthesia-related complications.

In the present study, the distribution of dental malocclusion classes was as follows: Class 2 (206 patients, 61.5%), Class 1 (93 patients, 27.8%), and Class 3 (36 patients, 10.7%). The mean age of the patients was 41 ± 15.69 years, and statistical analysis revealed no significant association between age and dental malocclusion class. Notably, Class 2 dental malocclusion was the most prevalent, affecting 61.5% of patients, with a mean age of 41.9 years. These findings align with previous research. A similar study we performed earlier [13] found no significant correlation between age and airway difficulties based on video laryngoscopy grade. Similarly, Sanyal et al.'s study [16] compared airway assessment using the Mallampati classification in an Indian population with a mean age of 43 years and found no association between age and difficult airway. However, contrasting evidence exists. A large-scale study (n=45,447) identified age ≥ 46 years and Mallampati classification 3 and 4 as factors associated with difficult intubation, suggesting that good oral health may reduce the likelihood of difficult ventilation. This highlights the importance of oral health in airway management, as mask ventilation is generally more suitable for individuals with good oral health [17].

Further supporting the study’s focus on anatomical predictors, a study revealed that patients experiencing difficult laryngoscopy, characterized by a Cormack-Lehane grade of 3 or 4, exhibited notable differences in baseline traits such as inter-incisor gap, thyromental distance, and the presence of skeletal malocclusion [18]. Additionally, research by Hansen et al. [19] revealed that children with Class II malocclusion and a pronounced horizontal maxillary overjet exhibited notably smaller nasal airway dimensions and higher nasal resistance, indicating a potential link to sleep-disordered breathing. These results are consistent with the findings of the current study, reinforcing the connection between malocclusion and airway challenges.

Regarding gender distribution, the present study found that Class 2 dental occlusion was the most common in both men and women, with 133 men (35.5%) and 73 women (21.7%) affected. Overall, Classes 2 and 3 dental occlusions were more prevalent in men, and a statistically significant difference was found between gender and difficult airway. This aligns with previous studies, which have also reported differences in intubation difficulty between men and women. For example, our previous study [13] found that 21.24% of women and 16.5% of men experienced difficult intubation. Similarly, another study of 2254 patients found that men had more frequent airway problems and tracheal intubation difficulties than women, particularly those with Mallampati classification 2 and 3 [20]. These findings underscore the influence of gender on airway management outcomes.

This study revealed that 114 patients (with laryngoscopy grades 3 and 4) experienced airway management difficulties. Specifically, this study investigated the relationship between dental malocclusion class and video laryngoscopy grade. Among patients with laryngoscopy grades 3 and 4, Class 2 malocclusion was the most common. Furthermore, it was found that as the video laryngoscopy grade increased, the likelihood of encountering dental malocclusion in higher classes (2 and 3) also increased. These findings are consistent with a 2017 study that investigated the prediction of difficult laryngoscopy in patients undergoing coronary artery bypass grafting, which found that 10% of 345 patients had difficult laryngoscopy with video laryngoscopy grades 3 and 4 and a TMD score<5 (14).

This study also explored the association between the Mallampati classification and dental malocclusion class. As previously mentioned, 285 patients (85.1%) had Mallampati class 2, 29 patients (8.7%) had Mallampati class 1, and 20 patients (6%) had Mallampati class 3, with Mallampati class 2 and 3 being more common in patients with dental malocclusion classes 2 and 3. However, the relationship between Mallampati classification and difficult airway management remains debated. Other studies that have examined the Mallampati classification in this context have reported varying results. For example, Mohammadi et al. found no significant association between the Mallampati classification and the PE/E-VC ratio (15). In contrast, studies of patients with difficult intubation have found that Mallampati class 2 and 3 were present in 80-88% of cases [21]. Given these inconsistencies, researchers have recently proposed a new classification system based on the direct visualization of the vocal folds using a laryngoscope, which may provide a more accurate prediction of difficult intubation [22].

Additionally, there is a notable discrepancy between Mallampati class II and III in the classification systems. Despite this, other studies have found the Mallampati classification to be useful in predicting difficult intubation [23]. This highlights the need for further research to standardize and improve airway assessment tools.

## Conclusion

In general, in the present study, there was no difference between dental malocclusion class and demographic indicators, including height, weight, and age. Nevertheless, dental malocclusion was more prevalent in men than women in all three classes, and a significant difference was seen in the second class of dental malocclusion. Also, in the present study, it was found that a significant relationship was observed between the class of dental malocclusion in patients and the indicators of the video laryngoscope grade and the degree of malampathi, which can be inferred that the high grades of the laryngoscope and malampathi criteria are related to class 3 and 2 of dental occlusion. Therefore, they are important in predicting and managing airway problems. It is suggested that in the future studies, more and more in-depth studies be done on the consequences of this difference in viewpoint and effective educational interventions to reduce the difference between the viewpoints of faculty members and students are scientifically extracted.

## Conflict of Interest

The authors declare that there is no conflict of interest associated with this research.
